# Supplementary material for: WNT Activation and TGFβ-Smad Inhibition Potentiate Stemness of Mammalian Auditory Neuroprogenitors for High-Throughput Generation of Functional Auditory Neurons In Vitro
Source: Cells. 2022 Aug 5;11(15):2431. doi: 10.3390/cells11152431 (PMC9367963; doi:10.3390/cells11152431)
Supplement: Supplementary file 1 [file cells-11-02431-s001.zip › cells-1794409-supplementary.pdf]

Supplementary Figure S1

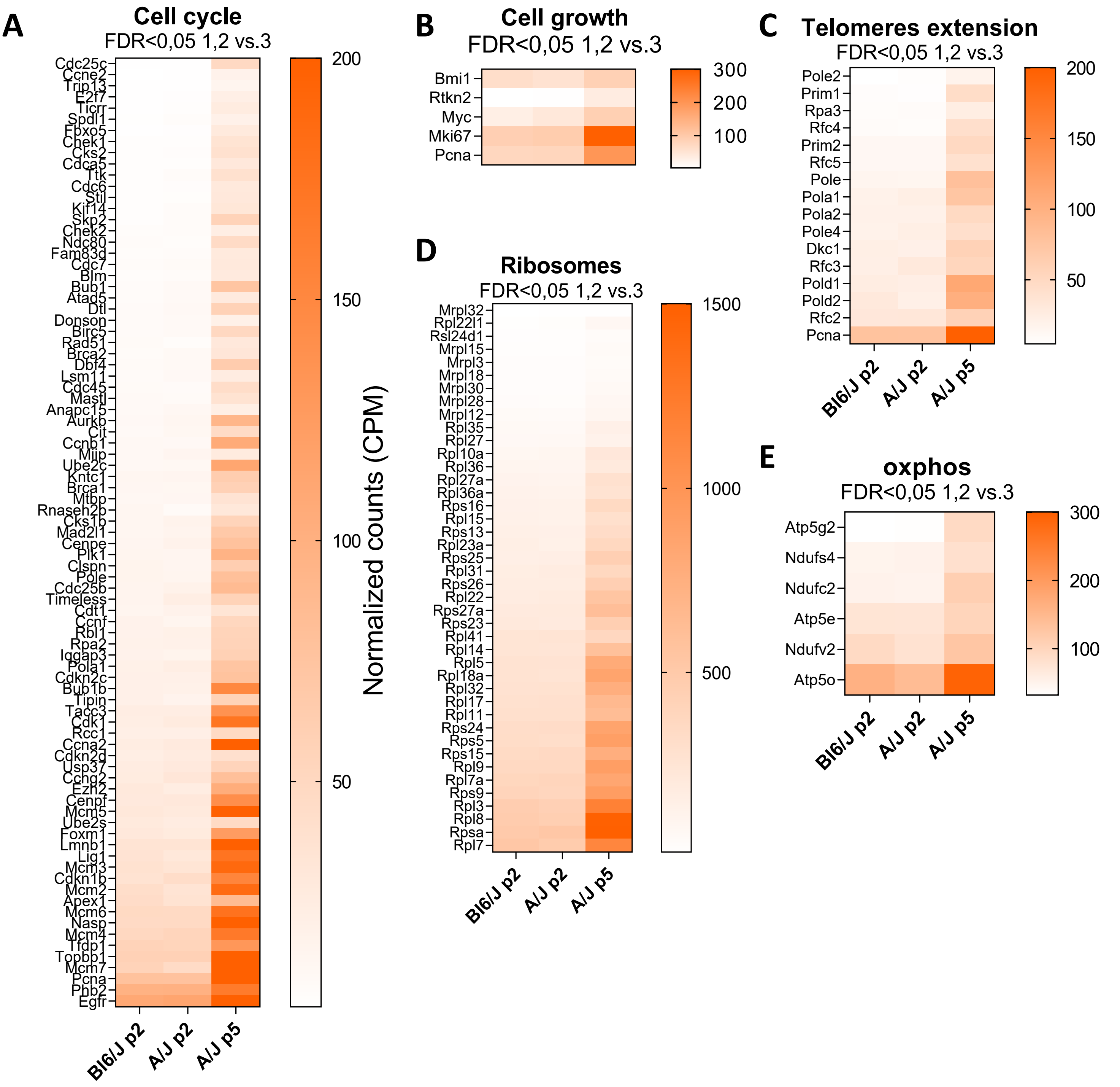

Supplementary Figure S1. Heatmaps showing the relative expression level of genes belonging to the cell cycle (**A**), cell growth (**B**), telomeres extension (**C**), Ribosomes (**D**) and oxidative phosphorylation (**E**) ontologies, in low stemness ANPGs from C57Bl/6 at passage 2 (lane 1), A/J at passage 2 (lane 2) and high stemness A/J ANPGs at passage 5 (phoenix) (lane 3). All genes displayed are significantly differentially expressed (FDR<5%) between low stemness (C57Bl/6 and A/J ANPGs) and phoenix ANPGs.

Supplementary Figure S2

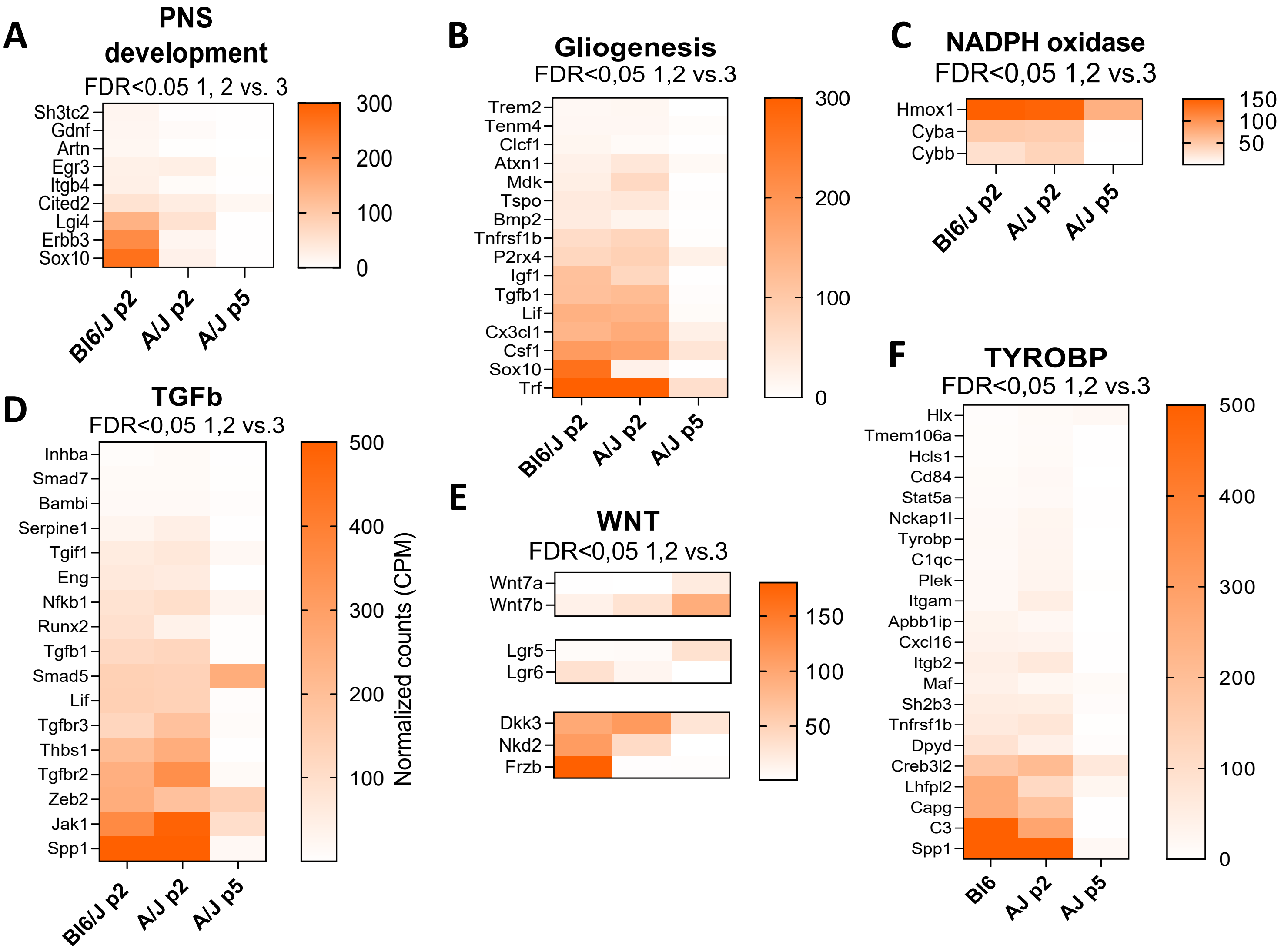

Supplementary Figure S2. Heatmaps showing the relative expression level of genes related to the peripheral nervous system development (**A**), gliogenesis (**B**), NADPH oxidase (**C**), TGFβ pathway (**D**), WNT pathway (**E**) and TYROBP pathway (**F**) in low stemness ANPGs from C57Bl/6 at passage 2 (lane 1) and A/J at passage 2 (lane 2) and high stemness A/J ANPGs at passage 5 (lane 3). All genes displayed are significantly differentially expressed (FDR<5%) between low stemness (C57Bl/6 and A/J ANPGs) and phoenix ANPGs.

Supplementary Figure S3

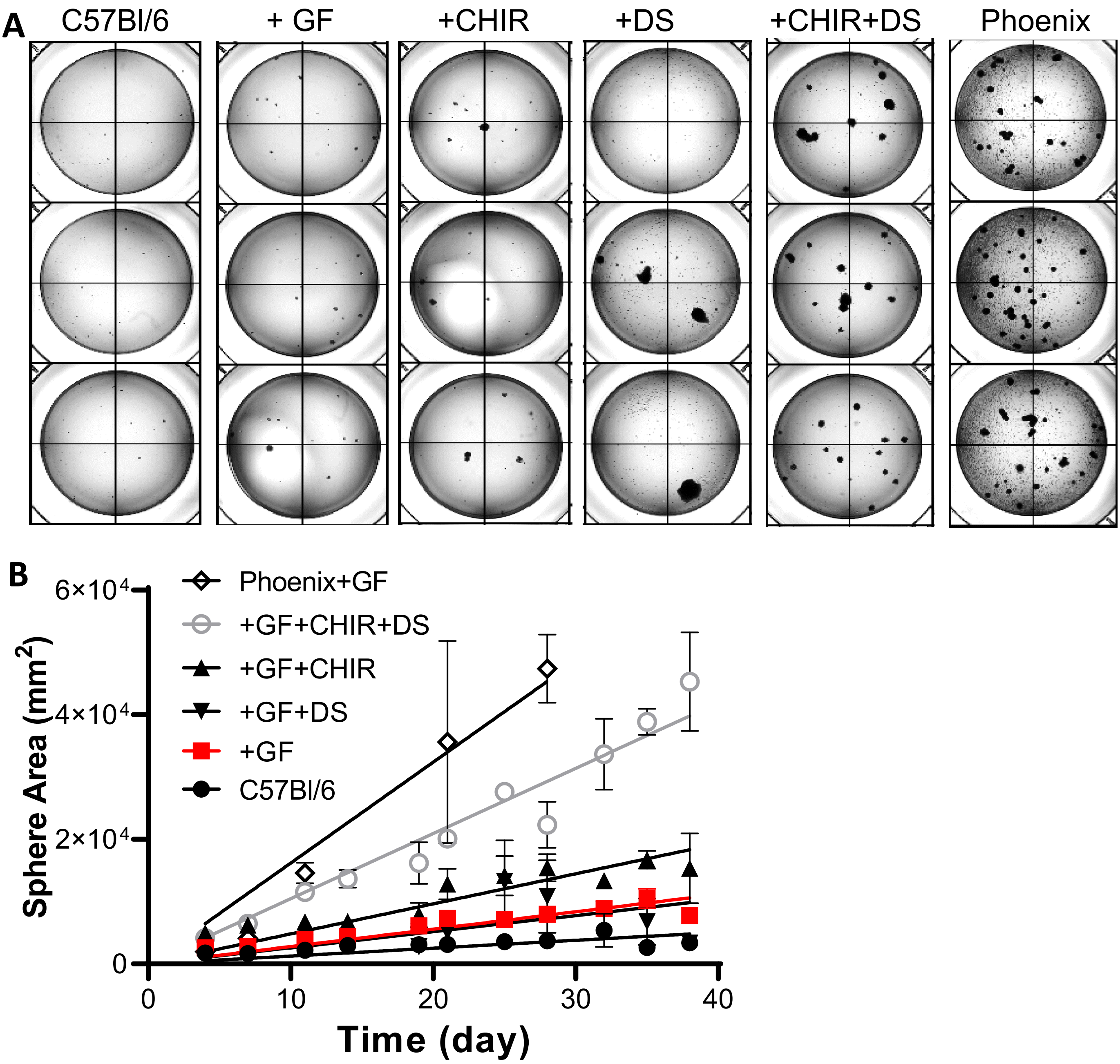

Supplementary Figure S3. A) A) C57Bl/6 ANPG were plated at 10'000/ well of a 96 well plate and treated with WNT agonist (CHIR99021; 3µM) and / or TGFβ Smad antagonist (dual SMAD inhibitors; LDN193189 0,5µM, SB431542 10µM) aiming at replicating phoenix ANPG pattern of gene expression and subsequent stemness phenotype. Pictures showing representative triplicate of ANPG spheres cultured for 1 month in a 96 well plate. Phoenix cells are used as positive control. C57Bl/6: ANPGs were cultured in DMEM:F12+N2 and B27 supplements without growth factors. +GF: DMEM:F12+N2 and B27 +IGF +EFG +HS +FGF (previously described conditions). GF+DS:DMEM:F12+N2 and B27 +IGF +EFG +HS +FGF +LDN193189 0,5µM +SB431542 10µM. GF+CHIR: DMEM:F12+N2 and B27 +IGF +EFG +HS +FGF +CHIR99021 3µM. +GF+CHIR+DS: DMEM:F12+N2 and B27 +IGF +EFG +HS +FGF +LDN193189 0,5µM +SB431542 10µM +CHIR99021 3µM. Phoenix +GF: phoenix ANPGs cultured in standard conditions (DMEM:F12+N2 and B27 + IGF + EFG + HS + FGF). B) Graph showing the average sphere area at different time points (up to 38 days). By both enhancing WNT pathway and repressing the TGFβ Smad pathway, we were able to enhance dramatically growth of low stemness C57Bl/6 ANPGs (grey empty circles) compared to previous "state of the art" conditions (highlighted in red), to a level approaching phoenix ANPGs (black lozenges). Data represent the average ± SEM of three independent experiments.

Supplementary Figure S4

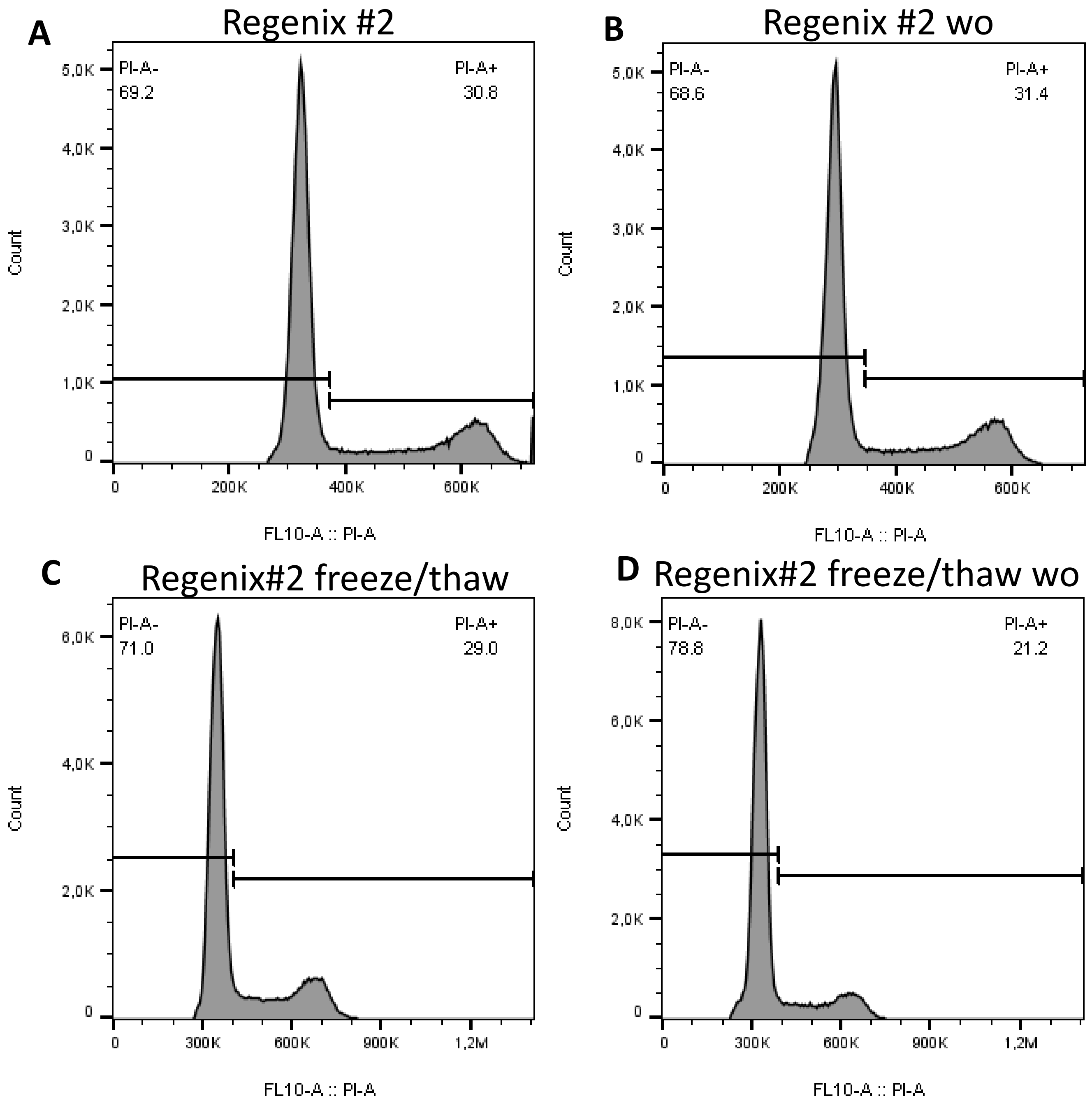

Supplementary Figure S4. Neither freeze / thaw cycles nor removal of reprogramming factors affect proliferation of stemness-induced C57Bl/6 ANPGs. Following DNA staining, flow cytometry was performed to determine the percentage of cells engaged in the cell cycle (phase S/G2M; proliferating) in reprogrammed C57Bl/6 ANPGs at passage 20 (**A**). At passage 20 after 2 weeks removal of WNT agonist (CHIR99021; 3µM) or TGFβ smad antagonist (dual SMAD inhibitors; LDN193189 0,5µM, SB431542 10µM) treatment (**B**). At passage 20 following one freeze and thaw cycle (**C**). And at passage 20 following one freeze and thaw cycle after 2 weeks removal of WNT agonist (CHIR99021; 3µM) or TGFβ smad antagonist (dual SMAD inhibitors; LDN193189 0,5µM, SB431542 10µM) treatment (**D**). In any condition, a significant amount of progenitor cells are proliferating, demonstrating that stemness-induced ANPGs, like phoenix cells, can survive to freeze / thaw cycles and that reprogramming is long lasting, even following reprogramming factors withdrawal.

Supplementary Figure S5

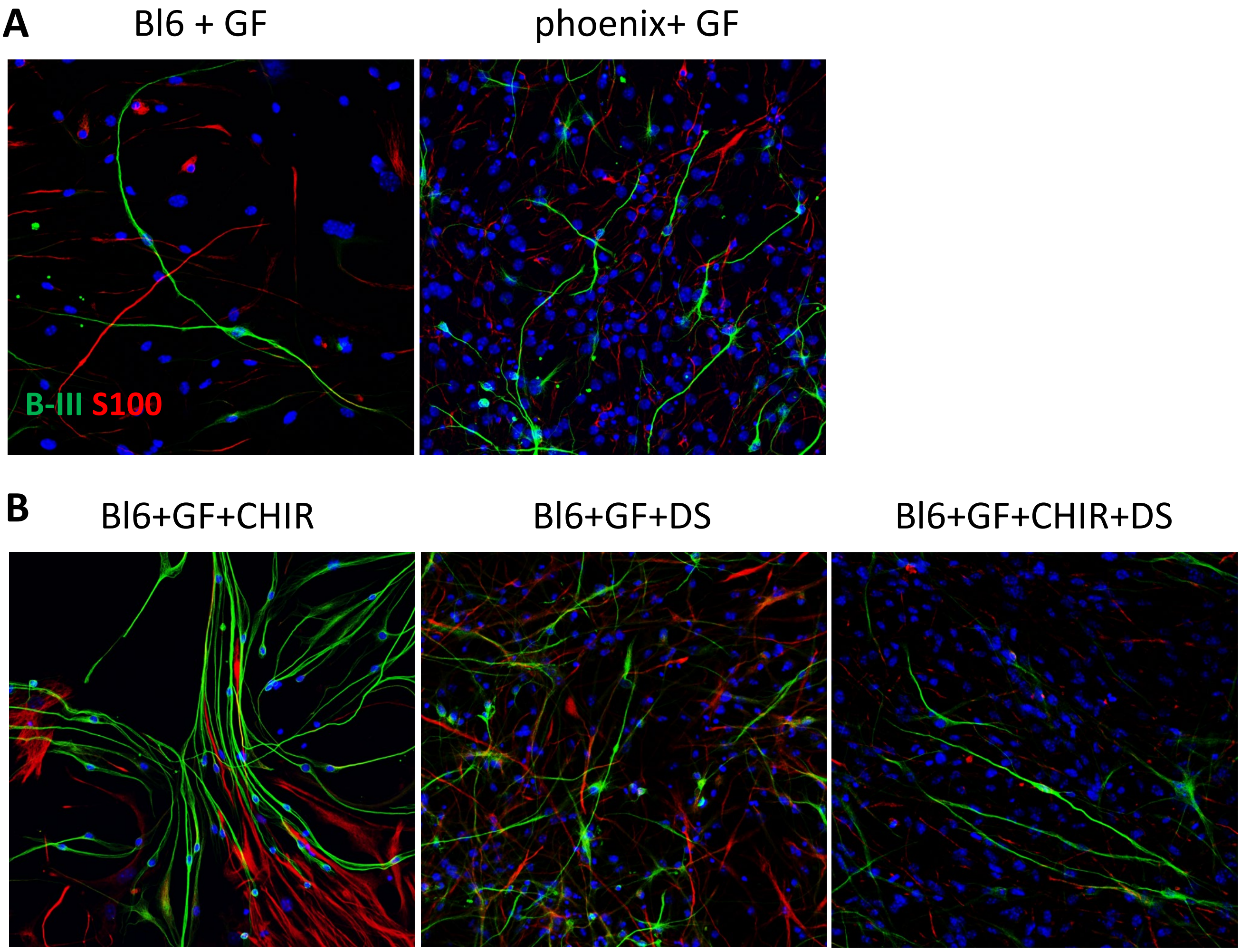

Supplementary Figure S5. Neurogenic potential of stemness-induced ANPGs. After treatment with WNT agonist (CHIR99021; 3µM) and / or TGFβ Smad antagonist (dual SMAD inhibitors; LDN193189 0,5µM, SB431542 10µM), ANPGs were differentiated on Matrigel coating following removal of mitogenic factors. BDNF (10ng/mL), NT-3 (50ng/mL) and LIF (10ng/mL) were added during the 7 days of differentiation. Cells were fixed for immunostaining with BIII-tubulin (green) and S-100 (red), respectively staining neuron and glial cells. Representative pictures of differentiated C57Bl/6 (low stemness) and phoenix ANPGs, respectively used as negative and positive controls for stemness induction and of differentiated stemness-induced ANPGs. Stemness induction was induced by CHIR99021; 3µM(CHIR); dual SMAD inhibitors LDN193189 0.5µM and SB431542 10µM (DS) and both treatments together (CHIR+DS). In every cases, treated ANPGs were able to differentiate into neurons (expressing BIII-tubulin) and glial cells (expressing S-100).

Supplementary Figure S6

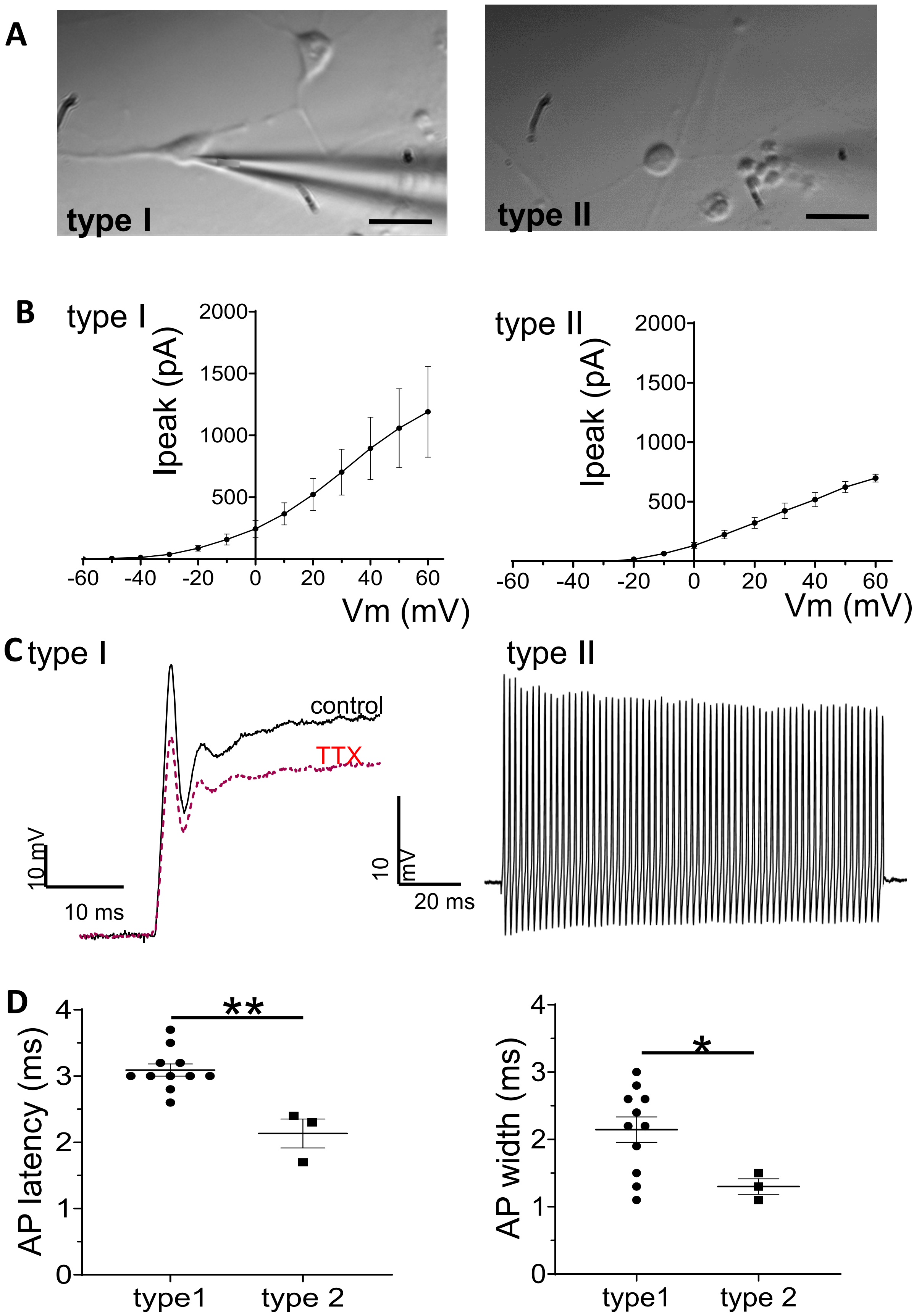

Supplementary Figure S6: Two different neuronal classes in the differentiated phoenix cell line. A. Photomicrographs under DIC contrast of type I and type II cells approached by the patch pipette from the right. Type I cells have a longitudinal soma whereas type II cells have a more spherical soma. Scale bars 10 µm. B. Current-voltage relationships of potassium currents. Type I cell potassium currents have a more negative reversal potential (-43.5 ± 3.1 mV, n=5) compared to type II cells (-24.9 ± 1.5 mV, n=3) and a more pronounced potassium currents (1190.2 ± 367.3 pA, n=5) than type II cells (627.3 ± 73.4 pA, n=3). C. Phoenix cells express voltage-gated sodium channels that are efficiently blocked by TTX (left), leading to sustained action potential firing in type II cells (right). D. Action potential latency and action potential width are significantly decreased in type II cells in accordance with their sustained firing properties.

Supplementary Figure S7

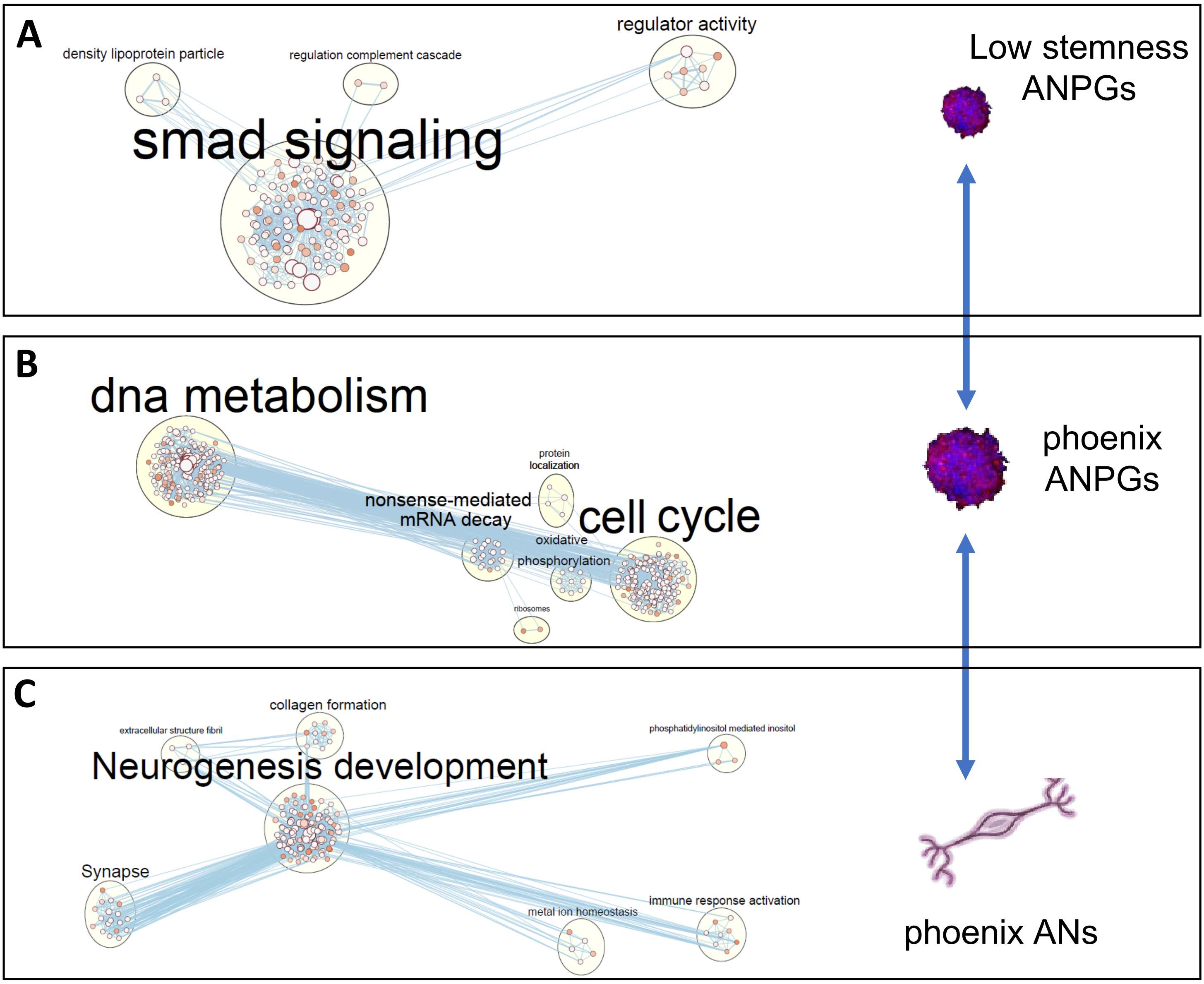

Supplementary Figure S7. Gene ontology network of low propagation ANPGs and phoenix ANPGs and derived neurons. Networks built from differentially regulated gene ontologies between low stemness (C57Bl/6 and A/J p2), high stemness (A/J p5) and derived auditory neurons. Each node represents a GO term, edges are drawn when there are shared genes between two GO terms.

Supplementary Table S1

Antibody	reference	species	dilution
Beta 3 tubulin (TUJ1)	Biolegend 802001	rabbit	1/2000
Beta 3 tubulin (TUJ1)	Sigma<br>T8660	mouse	1/1000
Beta 3 tubulin (TUJ1)	Biolegend 801202	mouse	1/1000
Ki67	Abcam ab16667	rabbit	1/100
Nestin	Chemicon<br>MAB353	mouse	1/2500
S100	Sigma<br>S2532-100UL	mouse	1/500
SOX2 E4	Santa Cruz<br>sc365823	mouse	1/100

Supplementary Table S1. List of antibodies used in the study.
